# Convergent Effects of Resveratrol and PYK2 on Prostate Cells

**DOI:** 10.3390/ijms17091542

**Published:** 2016-09-13

**Authors:** Andrea Conte, Annamaria Kisslinger, Claudio Procaccini, Simona Paladino, Olimpia Oliviero, Francesca de Amicis, Deriggio Faicchia, Dominga Fasano, Marilena Caputo, Giuseppe Matarese, Giovanna Maria Pierantoni, Donatella Tramontano

**Affiliations:** 1 Department of Molecular Medicine and Medical Biotechnologies, University of Naples “Federico II”, 80131 Naples, Italy; andrea.conte@unina.it (A.C.); spaladin@unina.it (S.P.); dominga.fasano@gmail.com (D.F.); marilenacaputo86@gmail.com (M.C.); giuseppe.matarese@unina.it (G.M.); 2 Institute of Experimental Oncology and Endocrinology, National Research Council of Italy, 80131 Naples, Italy; a.kisslinger@ieos.cnr.it (A.K.); claudio.procaccini@cnr.it (C.P.); 3 Centro di Ingegneria Genetica (CEINGE)—Biotecnologie Avanzate, 80131 Naples, Italy; 4 Institute of Polymers, Composites and Biomaterials, National Research Council of Italy, 80131 Naples, Italy; olivier@unina.it; 5 Centro Sanitario, University of Calabria, 87036 Rende (CS), Italy; francesca.deamicis@unical.it; 6 Department of Pharmacy, Health Science and Nutrition, University of Calabria, 87036 Rende (CS), Italy; 7 Department of Medical and Translational Science, University Federico II of Naples, 80131 Naples, Italy; d.faicchia@libero.it

**Keywords:** prostate cells, proline-rich tyrosine kinase 2 (PYK2), resveratrol, reactive oxygen species (ROS), autophagy, cell morphology, cell size

## Abstract

Resveratrol, a dietary polyphenol, is under consideration as chemopreventive and chemotherapeutic agent for several diseases, including cancer. However, its mechanisms of action and its effects on non-tumor cells, fundamental to understand its real efficacy as chemopreventive agent, remain largely unknown. Proline-rich tyrosine kinase 2 (PYK2), a non-receptor tyrosine kinase acting as signaling mediator of different stimuli, behaves as tumor-suppressor in prostate. Since, PYK2 and RSV share several fields of interaction, including oxidative stress, we have investigated their functional relationship in human non-transformed prostate EPN cells and in their tumor-prone counterpart EPN-PKM, expressing a PYK2 dead-kinase mutant. We show that RSV has a strong biological activity in both cell lines, decreasing ROS production, inducing morphological changes and reversible growth arrest, and activating autophagy but not apoptosis. Interestingly, the PYK2 mutant increases basal ROS and autophagy levels, and modulates the intensity of RSV effects. In particular, the anti-oxidant effect of RSV is more potent in EPN than in EPN-PKM, whereas its anti-proliferative and pro-autophagic effects are more significant in EPN-PKM. Consistently, PYK2 depletion by RNAi replicates the effects of the PKM mutant. Taken together, our results reveal that PYK2 and RSV act on common cellular pathways and suggest that RSV effects on prostate cells may depend on mutational-state or expression levels of PYK2 that emerges as a possible mediator of RSV mechanisms of action. Moreover, the observation that resveratrol effects are reversible and not associated to apoptosis in tumor-prone EPN-PKM cells suggests caution for its use in humans.

## 1. Introduction

Prostate adenocarcinoma is the most common malignancy in men, and the second leading cause of cancer-related deaths [1]. A strong connection between this disease and pathological and life-style conditions, known to drive inflammation and oxidative stress, is supported by prostate cancer higher incidence in hypertensive patients and higher aggressiveness in obese people [2]. Moreover, high-fat diets increase oxidative stress, enhancing proliferation of prostate cancer cells, whereas diets rich in food antioxidants are reported to fight the detrimental effects of reactive oxygen species (ROS), and may play an important role in preventing disease onset and progression [3,4]. Oxidative stress is a key factor in the development of cancer, since cellular homeostasis depends on intracellular ROS levels. In fact, low and moderate levels of ROS, acting as signaling molecules, sustain cellular proliferation and differentiation, and activate stress-responsive survival pathways. Conversely, high concentrations of ROS damage cellular macromolecules and promote cell death. As result of alterations in cellular metabolism, cancer cells display elevated ROS levels compared to normal counterparts, and are able to develop powerful compensative mechanisms to counteract the detrimental effects of ROS overproduction [5,6]. Consistently, studies in archival prostate cancer specimens reveal that ROS levels are higher in cancerous than in non-cancerous cells [7], as well as intracellular H_2_O_2_ levels are higher in highly tumorigenic sublines of LNCaP, C4-2 and C4-2B cells than non-tumor cells [7]. In this picture, food antioxidans are under consideration for their potential role as chemo-preventive and/or therapeutic agents for prostate cancer.

Among dietary antioxidants, resveratrol (RSV), a polyphenolic phytoalexin from grapes and other fruits, has gained enormous attention because of its wide-ranging biological activities and remarkable clinical potential. In particular, beside its anti-oxidant effect, RSV mimics caloric restriction and displays anti-inflammatory, growth inhibiting and pro-apoptotic activities. The wide range of RSV effects provides a rationale for its potential benefits as chemo-preventive and/or therapeutic agent against cancer, cardiovascular, inflammatory, and neuro-degenerative diseases. However, RSV’s wide variety of pharmacologic activities is dramatically influenced by cellular context, dose, concentration, and duration of treatment, resulting in different or even opposite effects in different cell types [8,9,10,11]. Therefore, it is very difficult to understand how the same drug can affect so many different diseases [12,13,14] and which effect/s is/are relevant for human health. Although data from epidemiological and clinical studies do not justify the recommendation of RSV consumption by humans for any given indication, RSV enriched OTC (Over The Counter) products are available worldwide, and are suggested as health promoting and preventive tools for several diseases [15]. This raises the crucial issue of the long-term effects of this molecule, in particular on normal tissues. In fact, to exploit its chemo-preventive and protective potential, RSV should be administered chronically to healthy people and should interact with normal cells to prevent cell damage and, possibly, killing pre-cancerous cells. Although studies in humans suggest that RSV should be generally considered safe, high doses of RSV can cause gastrointestinal side effects and inhibit cytochrome P450 enzymes, increasing the risk of adverse effects of certain drugs [16]. RSV also exhibits estrogen-like properties and activates estrogen and androgen receptors-mediated transcription that can lead to the stimulation of cancer cell proliferation [16]. Moreover, several cell types like endothelial cells, lymphocytes, adipocytes, neurons, osteoblasts, hepatic cells, and epidermal keratinocytes are reported to be vulnerable to RSV [16,17,18,19,20,21,22]. Surprisingly, although the selective “killing” effect of RSV on tumor or transformed cells has generated a notable degree of expectation and interest on its use as anti-cancer drug, in most studies on cancer cells the normal cell counterpart is missing. Thus, whether RSV can cause damage to normal cells on the long run remains a serious question.

To contribute to this important issue, we have investigated the effects of RSV on non-transformed human cells. We have already reported that RSV enhances the detrimental effects of UVB in HaCat cells, a well-known model of non-transformed keratinocytes in culture [23,24]. Moreover, we reported that RSV inhibits cell adhesion and migration and activates Shc proteins in EPN cells [25,26], a line of non-transformed human prostate epithelial cells in culture, and in their tumor-prone counterpart bearing a kinase dead mutant of the proline-rich tyrosine kinase (PYK2), a non-receptor kinase of the focal adhesion kinase (FAK) family [27,28,29]. In addition, we have previously reported that PYK2 expression inversely correlates with degree of malignancy of prostate cancer cells [30] and that expression of a PYK2 dead kinase mutant (PKM) in prostate epithelial EPN cell line induces cell motility, migration and a massive reorganization of cytoskeleton [31,32].

Interestingly, PYK2 and RSV share several fields of interaction, such as oxidative stress, calcium, proliferation and cytoskeleton organization. In particular, RSV reduces ROS production, induces intracellular calcium mobilization, and binds αvß3 integrin [33,34], whereas PYK2 is an oxidative stress and calcium-regulated kinase, and interacts with the same integrin [35,36,37].

On the basis of the above-mentioned information, we aim to investigate the possible functional interaction between RSV and PYK2 in the regulation of prostate cells functioning. Here we show that RSV induces reversible growth arrest associated to morphological changes but not apoptosis in both EPN and EPN-PKM cells, whereas it strongly decreases cell number and induces apoptosis in PC3 cells, a well-known model of prostate cancer. Moreover, we found that PYK2 kinase mutation increases basal level of ROS production and of autophagy and modifies the intensity of the anti-oxidant and pro-autophagic effects of RSV in the EPN/EPN-PKM cell system. In addition, PYK2 knock-down by RNAi replicates the effects of the dead kinase in EPN cells, confirming the ability of PYK2 to regulate ROS production, autophagy levels, and sensitivity to RSV effects. Taken together, these data strengthen the role of PYK2 as a key regulator of prostate cells and suggest that PYK2 may be involved in the regulation of the intracellular redox state and of autophagy, two important functions by which RSV regulates cell fate. The findings that RSV has a strong bioactivity on both EPN and EPN-PKM cells, does not induce cell death in the tumor prone EPN-PKM cells, and has reversible effects also on cancer cells, once again suggests caution in a “too easy” use of this phytoalexin.

## 2. Results

### 2.1. The EPN, EPN-PKM and PC3 Cells Model

**Figure 1:**
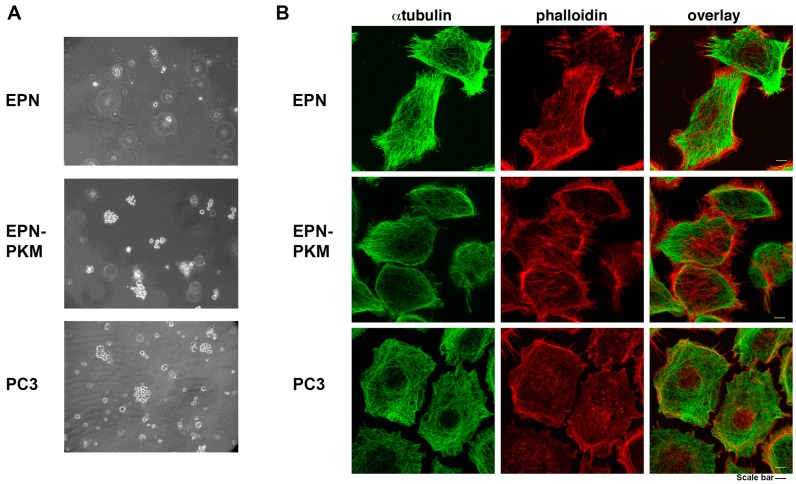
Growth in semi-solid medium and cytoskeleton organization of EPN, EPN-PKM and PC3 cells: (**A**) Growth of EPN, EPN-PKM and PC3 cells in semi-solid medium. EPN, EPN-PKM and PC3 cells single cell suspensions were seeded in agar-containing medium, observed daily and photographed after 15 days. Cells have been observed with Axiovert 25 (Carl Zeiss, Jena, Germany) and photographed with Canon GC5 (Canon Italia S.p.A, 20063 Cernusco sul Naviglio, Milan, Italy) (final magnification 40×). PC3 cells were used as positive controls; (**B**) Microtubule and Actin cytoskeleton morphology in EPN, EPN-PKM and PC3 cells. Cells were stained for double-immunofluorescence with a monoclonal antibody against α-tubulin revealed with a FITC-conjugated secondary antibody (**green**) and with TRITC-phalloidin (**red**). Bars, 5 μm.

To investigate the functional interaction between PYK2 and RSV, and to analyze the effects of RSV on non-transformed prostate cells, we selected as experimental models the non-transformed prostate epithelial cells EPN and their counterpart expressing the PKM kinase-dead mutant of PYK2 (EPN-PKM). To compare some effects induced by RSV on non-transformed cells to those induced on cancer cells, we used also PC3 cells, a well-known model of prostate cancer, derived from a bone metastasis of Glaeson VI grade adenocarcinoma. We have previously demonstrated that loss of PYK2 kinase activity confers tumor-like characteristics to epithelial prostate cells, such as a motile and migratory phenotype [25,30,31,32]. Consistently, as shown in Figure 1A, EPN-PKM, like PC3 cells, are able to grow in semisolid medium, whereas wild type EPN cells remain unable to do so [25]. In addition, EPN-PKM3 cells display a rearrangement of actin cytoskeleton, characterized by a significant increase in filopodia (Figure 1B). Moreover, while in EPN cell the microtubule network is very well defined, it appears defective in PC3 cells. Apparently, EPN-PKM cells resemble PC3 cells in terms of shape, filopodia and cortical actin, while they fall in between wild type EPN and PC3 cells as for stress fibers and microtubules organization.

### 2.2. The Effects of PYK2 Mutation and RSV on Basal and H_2_O_2_-Induced ROS Production in EPN and EPN-PKM Cells

**Figure 2:**
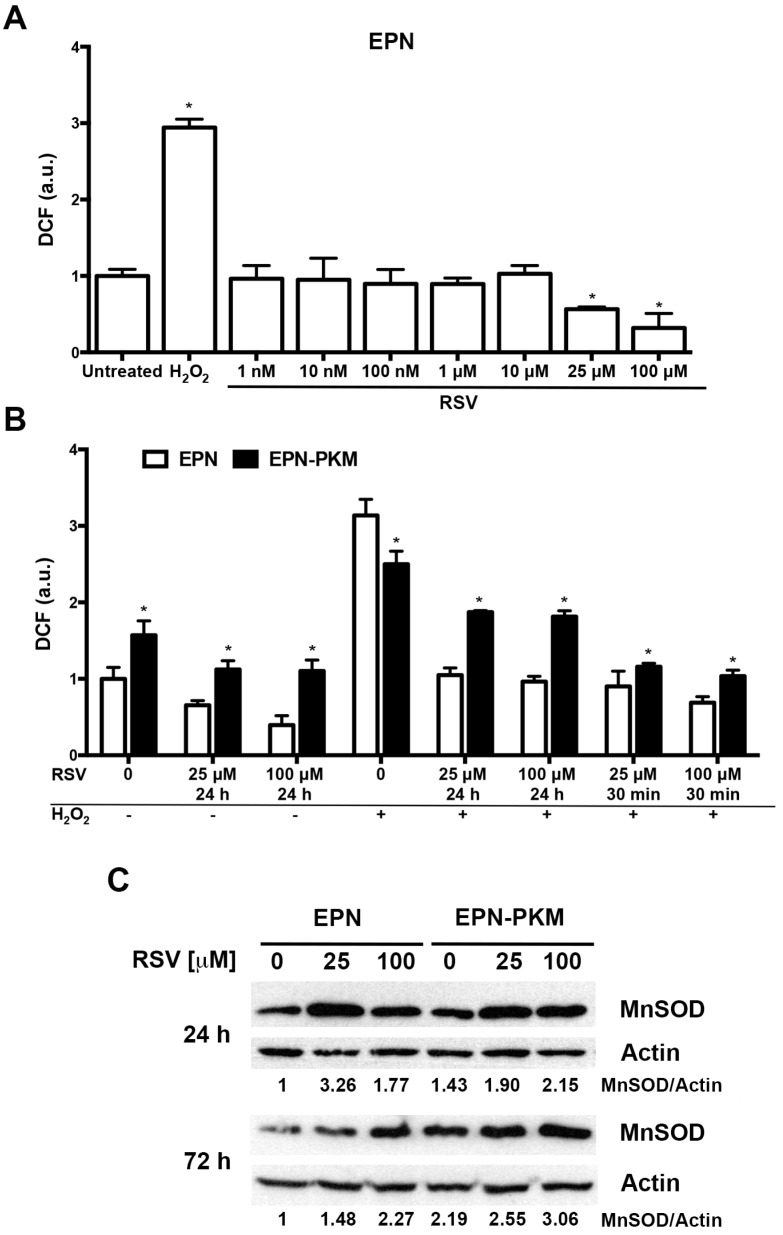
Effects of PYK2 mutation and RSV on ROS production and MnSOD expression in EPN and EPN-PKM cells: (**A**) ROS levels were estimated indirectly by measuring the fluorescence emitted by dichlorofluoresceine (DCF) 30 min after addition of RSV (1 nM to 100 µM) or 400 µM H_2_O_2_ to EPN cells. Data are mean ± SD of a representative experiment performed in triplicate. (*) indicates statistically significant differences (*p* < 0.05) between treated and untreated EPN cells; (**B**) EPN and EPN-PKM cells were either pretreated for 24 h with RSV (25 and 100 µM) prior to the addition of 400 µM H_2_O_2_, or treated concomitantly with RSV (25 and 100 µM) and 400 µM H_2_O_2_. The levels of ROS were estimated indirectly by measuring the fluorescence emitted by DCF 30 min after addition of H_2_O_2_. Data are mean ± SD of a representative experiment performed in triplicate. (*) indicates statistically significant differences (*p* < 0.05) between EPN and EPN-PKM cells at each experimental point; (**C**) Western blot analysis of MnSOD protein expression in EPN and EPN-PKM cells in control conditions and treated with 25 and 100 µM RSV for 24 or 72 h. β-Actin was used as loading control. The relative optical density of MnSOD levels normalized to β-Actin levels is shown.

The relationship between RSV and oxidative stress has been extensively studied, and the antioxidant activity of RSV, due to both direct scavenging and induction of phase II anti-oxidant enzymes, such as SOD and catalase [38,39], is one of its most important effects. However, at low concentration, and depending on cellular context, RSV may also behave as pro-oxidant agent [40,41]. To test whether RSV has anti-oxidant and/or pro-oxidant activity in EPN cells, we measured ROS levels 30 min after RSV addition (1 nM to 100 µM), using H_2_O_2_ (400 µM) as positive control. RSV decreases basal ROS levels in a dose-dependent fashion and did not show pro-oxidant effect at any concentration tested (Figure 2A). On this basis, and considering that PYK2 is an oxidative-sensitive kinase, we have evaluated the impact of RSV pre-treatment on basal and H_2_O_2_-induced ROS production in EPN and EPN-PKM cells. To this aim, RSV (25 and 100 µM) was added to EPN and EPN-PKM cells 24 h prior addition of 400 µM H_2_O_2_. As shown in Figure 2B, EPN-PKM cells display a basal ROS levels significantly higher than the parental EPN cells, indicating that PYK2 may be involved in the regulation of ROS production. Interestingly, H_2_O_2_-induced ROS formation was more potent in EPN than in EPN-PKM cells. Moreover, although RSV reduced basal and H_2_O_2_-induced ROS levels in both cell lines, its anti-oxidant effect was by far more potent in EPN than in EPN-PKM cells. Similar results have been obtained when RSV and H_2_O_2_ were conjointly added at time 0 and ROS levels measured 30 min thereafter (Figure 2B). To deepen the differences between EPN and EPN-PKM cells in basal ROS production and sensitivity to RSV anti-oxidant activity, we evaluated the expression of MnSOD after RSV treatment in both cell lines. As shown in Figure 2C, RSV increased MnSOD expression in EPN cells. Of note, consistently with their tumor-prone phenotype [42], EPN-PKM cells displayed significantly higher basal level of MnSOD than EPN cells, which was only moderately increased by RSV. Altogether, these data indicate that PYK2 is involved in the mechanisms regulating the sensitivity of prostate cells to redox intracellular state.

### 2.3. RSV Induces Growth Arrest and Morphological Changes in EPN, EPN-PKM and PC3 Cells

**Figure 3:**
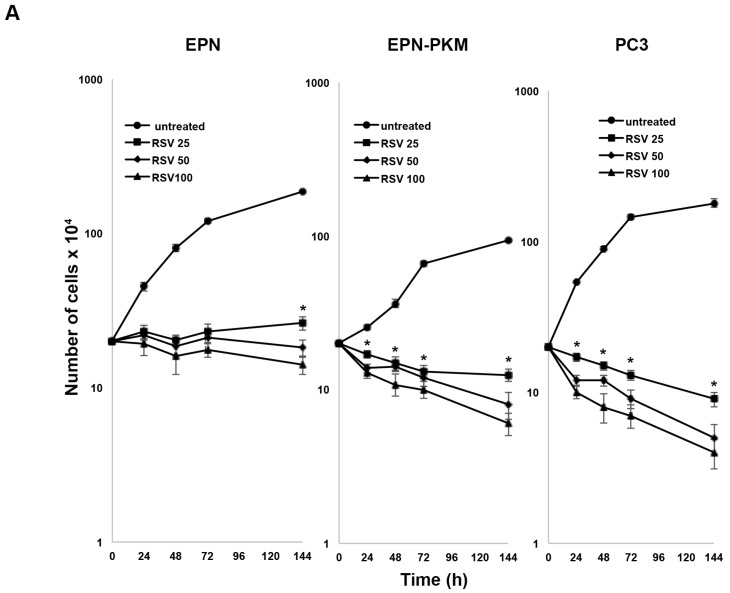

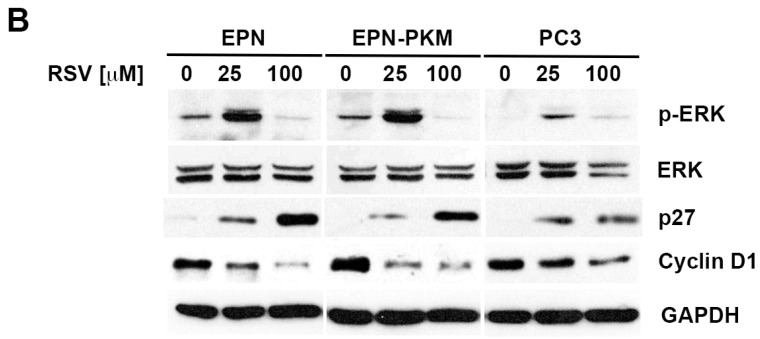
RSV induces growth arrest in EPN, EPN-PKM and PC3 cells: (**A**) RSV (25, 50 and 100 µM) was added 24 h after seeding 1 × 10^5^ EPN, EPN-PKM and PC3 cells in standard medium. Cell number was determined in a Neubauer hemocytometer at the indicated time points. Data are mean ± SD of a representative experiment performed in triplicate. The differences between treated and untreated cells are statistically significant (*p* < 0.05) for any tested concentration of RSV at any experimental time. (*) indicates statistically significant differences (*p* < 0.05) between cells treated with 25 µM RSV and those treated with 100 µM RSV; (**B**) Total cell lysates from EPN, EPN-PKM and PC3 cells in control conditions and treated with 25 and 100 µM RSV for 24 h were analyzed by Western blot using the indicated antibodies. GAPDH was used as loading control.

**Figure 4:**
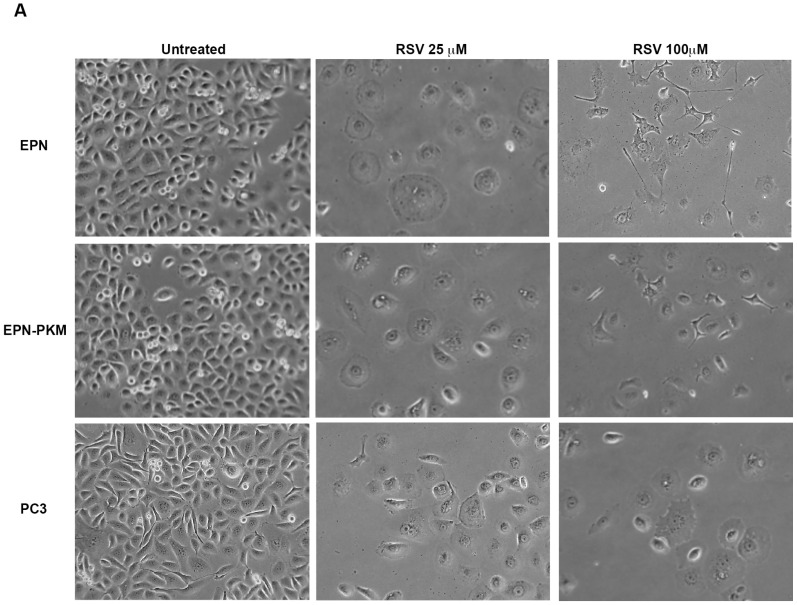

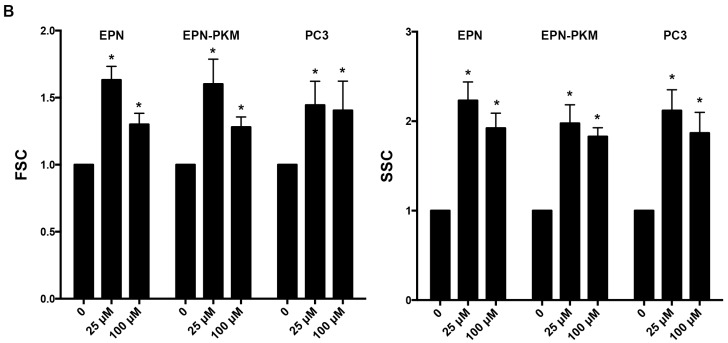
RSV alters shape and size of EPN, EPN-PKM and PC3 cells: (**A**) Microphotographs of representative fields of EPN, EPN-PKM and PC3 cells cultured in standard growing condition or in the presence of 25 and 100 µM RSV for 72 h. Cells have been observed with Axiovert 25 and photographed with Canon GC5 (final magnification 65×); (**B**) Relative size and internal complexity of EPN, EPN-PKM and PC3 cells in control conditions and treated with 25 and 100 µM RSV have been evaluated by flow cytometry, in terms of FSC and SSC respectively. (*) indicates statistically significant differences (*p* < 0.05) between treated and untreated EPN cells.

Since anti-proliferative activity of RSV is one of the major determinants of its anti-cancer potential, we investigated the effect of RSV treatment on the proliferation of EPN and EPN-PKM cells. As a control, we used also PC3 cells. Growth curve assay shows that 25, 50 and 100 µM RSV induces dose-dependent growth arrest in all the three cell lines. However, in both EPN-PKM and PC3 cells, prolonged treatment (four to seven days) with high doses of RSV (50 and 100 µM) reduces cell number, whereas in EPN cells number remained basically unchanged at all concentration tested, throughout the experimental period (Figure 3A). Therefore, we analyzed the expression and the activation of several markers of growth arrest, after 24 h of 25 and 100 µM RSV treatments (Figure 3B). In spite of its central role in cell proliferation, sustained and prolonged ERK activation has been also involved in growth arrest and programmed cell death [43,44]. In that, 25 µM RSV treatment induced a significant increase in ERK 1/2 phosphorylation in both EPN and EPN-PKM cells (Figure 3B), whereas 100 µM RSV induces a slightl decrease in ERK 1/2 activation. PC3 cells display low basal levels of ERK 1/2 phosphorylation, which were slightly increased by RSV treatment. Moreover, RSV decreases Cyclin D1 [45], and increases Cyclin-dependent kinase inhibitor 1B (p27^Kip1^) protein levels in EPN, EPN-PKM and PC3. Interestingly, RSV-induced growth arrest is associated to dramatic changes in cell shape in all three tested cell lines. Cells treated for 72 h with 25 µM RSV acquire a “sunny side up” appearance and their size consistently increases with respect to the untreated cells. Moreover, 100 µM RSV has a different effect on cellular morphology characterized by the appearance of an “astrocyte-like” shape in EPN and EPN-PKM cells (Figure 4A). To evaluate whether increase in cell size is due only to a spreading effect or represents a real increase in cellular volume, we analyzed cellular physical parameters by flow cytometry. In particular, FSC (Forward SCatter), measuring the amount of the laser beam that passes around the cell, gives cell relative size/volume, whereas SSC (Side SCatter), measuring the amount of laser beam that bounces off of particulates inside cells, gives relative level of cell granularity (i.e., complexity). As shown in Figure 4B, RSV treatment induces increase in both size and complexity in all the three lines tested. Consistently with morphological observation, increase in cell volume was more pronounced in 25 µM than in 100 µM in EPN and EPN-PKM treated cells, whereas no significant dose dependency was observed in PC3 cells.

**Figure A1:**
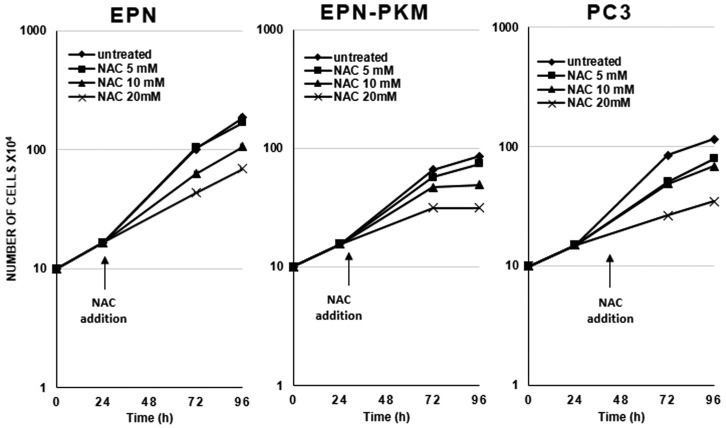
The effect of NAC on the proliferation of EPN, EPN-PKM and PC3 cells NAC (5, 10 and 20 mM) was added 24 h after seeding 1 × 10^5^ EPN, EPN-PKM and PC3 cells in standard medium. Cell number was determined in a Neubauer hemocytometer at the indicated time points. Data are mean ± SD of a representative experiment performed in triplicate.

Finally, as a control, we have also evaluated the impact on cellular proliferation of *N*-Acetyl-Cysteine (NAC), another well-known anti-oxidant agent [46]. As previously reported for other prostate cell lines [47], we have found that NAC, differently from RSV, only slows down proliferation of EPN, EPN-PKM and PC3 cells without inducing a growth arrest (see Figure A1 and Table A1). In addition, NAC treatment does not induce morphological changes, indicating that the observed changes in cell size and shape are specific effects of RSV.

### 2.4. EPN, EPN-PKM and PC3 Cells Proliferation Re-Starts upon Resveratrol Wash-out

**Figure 5:**
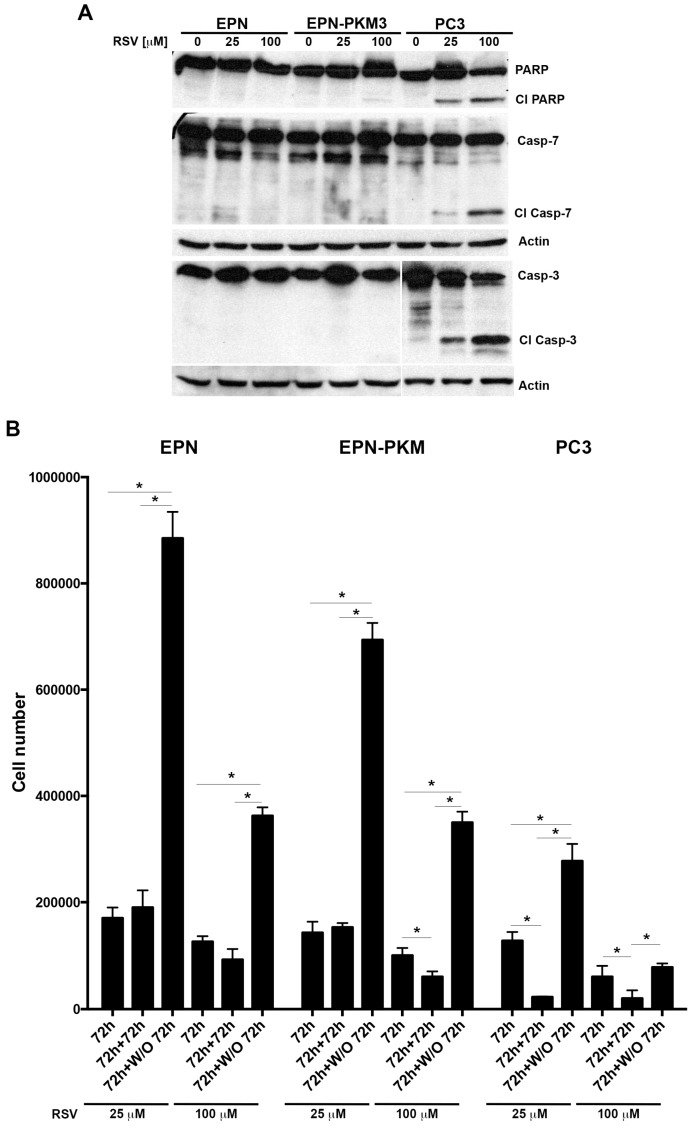
EPN, EPN-PKM and PC3 cells proliferation re-starts upon resveratrol wash-out (**A**) Western blot analysis of the effect of 72 h RSV treatment (25 and 100 µM) on the expression of native and cleaved form of PARP, caspase-7 and caspase-3 in EPN, EPN-PKM and PC3 cells. Actin was used as loading control; (**B**) RSV (25 and 100 µM) was added 24 h after seeding 1 × 10^5^ EPN, EPN-PKM and PC3 cells in standard medium. After 72 h, medium was replaced with RSV-containing medium in a group of dishes and with standard medium in another one. Cell number was determined in a Neubauer hemocytometer after 72 h of treatment (72 h), 144 h of treatment (72 h + 72 h) and 72 h of treatment followed by 72 h of W/O (72 h + W/O 72 h). Data are mean ± SD of a representative experiment performed in triplicate. (*) indicates statistically significant differences (*p* < 0.05) between each pair of experimental points connected by the horizontal bar.

**Figure 6:**
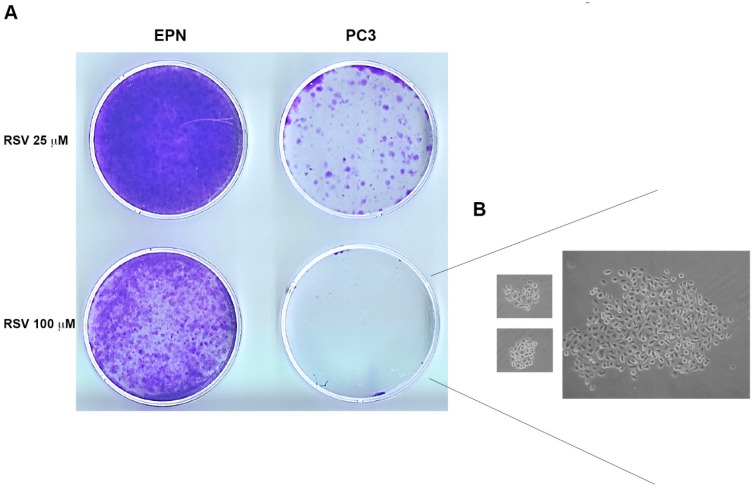
Colonies formed by EPN and PC3 cells post long-term RSV treatment followed by W/O: (**A**) Crystal Violet staining of cell colonies arising from EPN and PC3 cells treated with RSV (25 and 100 µM) for seven days followed by seven days W/O; and (**B**) microphotographs of representative colonies arising from PC3 cells treated with 100 µM RSV for seven days followed by seven days W/O. Cells have been observed with Axiovert 25 and photographed with Canon GC5 (final magnification 40×).

**Figure 7:**
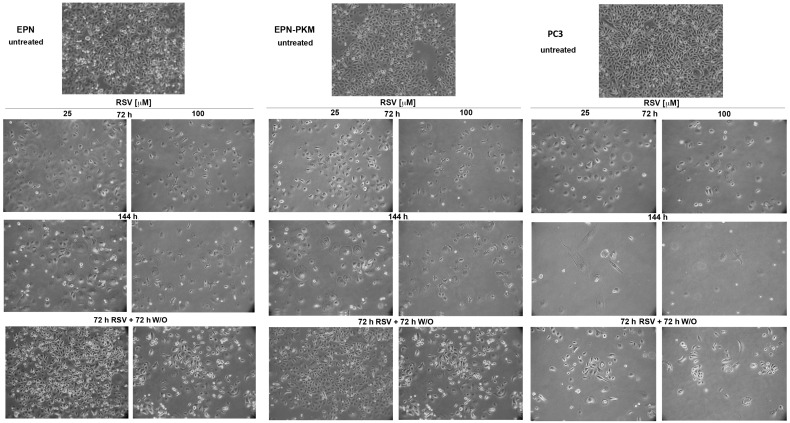
RSV effects on cellular morphology are reversible Microphotographs of representative fields of EPN, EPN-PKM and PC3 cells cultured in standard growing condition or in the presence of 25 and 100 µM RSV for 72 or 144 h, or cultured for 72 h in the presence of 25 and 100 µM RSV and for 72 h in its absence (72 h RSV + 72 h RSV W/O). Cells have been observed with Axiovert 25 and photographed with Canon GC5 (final magnification 40×).

Considering the differential effect of RSV on the proliferation of the three cell lines, to test whether it would exert a cytostatic or a cytotoxic effect depending on the cellular contest, we investigated whether RSV treatment is associated to apoptosis. Interestingly, as shown in Figure 5A, 72 h of RSV treatment, induces cleavage of Poly (ADP-ribose) polymerase (PARP), caspase-7 and caspase-3 in PC3 but not in EPN and EPN-PKM cells, confirming the knowledge that RSV induces apoptosis selectively in cancer cells. Since we and others reported that RSV effects on proliferation are reversible in some cell lines [23,48], we investigated this issue in prostate cells. To this aim, after 72 h treatment, we performed a RSV wash-out (W/O), followed by additional 72 h of culture. As shown in Figure 5B, the number of EPN and EPN-PKM cells increases after 25 and 100 µM RSV withdrawal, whereas it further decreases when the treatment with 100 µM RSV is prolonged for additional 72 h. More importantly, despite apoptosis activation, also PC3 cells, whose number was lower than that of EPN and EPN-PKM cells post-72 h of treatment, re-started to proliferate after 25 µM RSV W/O. Interestingly, after 100 µM RSV-W/O, PC3 cell number did not further decrease with respect to cells treated for 72 h. Noteworthy, the increase in cell number observed after RSV-W/O was consistently lower in PC3 than in EPN and EPN-PKM cells. To better evaluate the recover potential of PC3 cells, we analyzed cell growth also after seven days of RSV treatment followed by seven days W/O. Cristal Violet staining shows that PC3 cells treated with 25 µM RSV restarted proliferation after RSV removal (Figure 6A). In addition, microscopy analysis revealed that, also after 100 µM RSV-W/O, the few surviving PC3 cells form small clones, indicating that single cells recover and proliferate after long treatment with high doses of RSV (Figure 6B). Conversely, and in accord with the cytostatic effect exerted by RSV on EPN cells, seven days after RSV removal, EPN cells substantially form monolayers covering almost the whole surface of the culture dishes. Thus, prolonged treatment further enlights the different response to RSV of non-transformed and cancer cells. Finally, we noticed that also cell shape start to revert after RSV removal already after 24 h, and by 72 h the majority of the cells display morphology substantially similar to the untreated cells in all three lines (Figure 7).

Taken together, these data indicate that RSV induces reversible growth arrest not associated to apoptosis in non-tumor and tumor-prone prostate cells and that PC3 cancer cells are consistently more sensitive than EPN and EPN-PKM cells to its anti-proliferative and pro-apoptotic effects.

### 2.5. PYK2 Ablation Increases Basal and RSV-Induced Levels of Autophagy in EPN Cells

**Figure 8:**
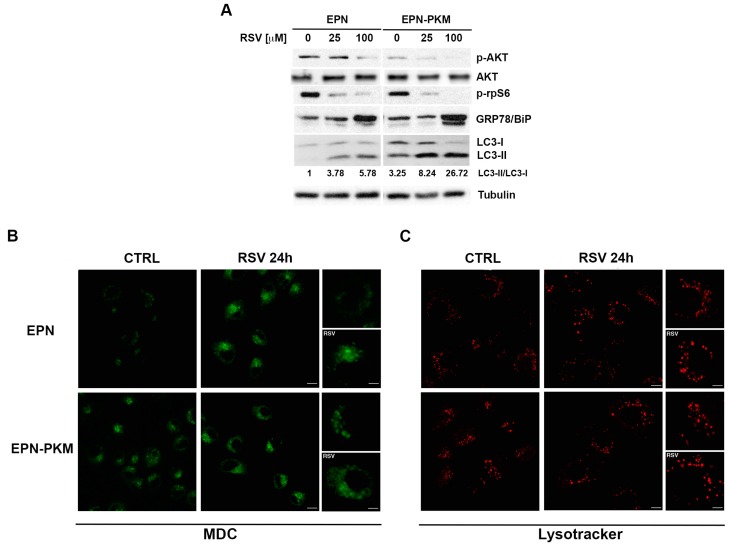
PYK2 and RSV affect autophagy in EPN and EPN-PKM cells: (**A**) Total cell lysates from EPN and EPN-PKM cells in control conditions or treated with 25 and 100 µM RSV for 24 h were analyzed by Western blot using the indicated antibodies. Tubulin was used as loading control. The relative optical density of LC3-II/LC3-I ratio is shown; (**B**,**C**) EPN and EPN-KM cells, in control conditions (CTRL) or treated with RSV for 24 h were stained with monodansylcadaverin (MDC, in green, **B**) or lysotracker (in red, **C**) as described in materials and methods. Serial confocal sections were collected for each condition, pictures at higher magnification are shown. Bars, 10 μm.

Since decrease in cell number observed in EPN-PKM cells was not associated to apoptosis, we explored autophagy as an alternative process of cell viability regulation. RSV displays pro-autophagy effect in several cellular contexts [24,49], thus we have investigated whether it could induce autophagy also in EPN cells and whether loss of PYK2 could influence this process. To this aim, we evaluated the phosphorylation of AKT, a well-known pro-survival marker, and of the ribosomal protein S6 (rpS6), the final read-out of mTORC1 cascade that, as well as AKT pathway, negatively regulates autophagy. RSV decreases phosphorylation of AKT and rpS6 in both EPN and EPN-PKM cells (Figure 8A). Moreover, RSV increases the expression of GRP78/BiP, a hallmark of ER stress often associated to autophagy [50]. Consistently, RSV increases in a dose-dependent manner LC3-I > LC3-II conversion rate, a hallmark of autophagy [51], enforcing the hypothesis that RSV treatment induces autophagy also in prostate cells (Figure 8A). Intriguingly, this analysis reveals that EPN-PKM cells displays a higher basal LC3-II/LC3-I ratio compared to EPN, indicating that PYK2 kinase activity may be involved in the regulation of autophagy. In addition, autophagy was more consistent in cells expressing the PYK2 dead kinase also after RSV treatment, suggesting that PYK2-deficient cells may be more sensitive to autophagic cell death. To gain insight into the autophagy-promoting action of RSV, we analyzed the presence and the distribution of autophagosomes and lysosomes in EPN and EPN-PKM cells. To this aim, we visualized autophagosomes in vivo by using the fluorescent dye mono-dansyl-cadaverine (MDC) [52], and in agreement with biochemical data, we found that in both cell lines RSV induces a strong increase of autophagosomes (Figure 8B). Moreover, at the steady-state EPN-PKM cells showed more autophagosomes (in terms of number and size) in comparison to EPN cells (compare upper and lower panels of Figure 8B), confirming that PYK2 kinase mutation increases autophagy. Consistently, in those conditions we also observed enlargement of the lysosome compartments (Figure 8C). Altogether, these data indicate that RSV induces the activation of the autophagy pathway in prostate cells and suggest that PYK2 deficiency may result in increased susceptibility to RSV-induces autophagy cell death.

### 2.6. PYK2 Knock-down Confirms the Involvement of the Kinase in the Regulation of ROS Production, Autophagy and Sensitivity to RSV in EPN Cells

**Figure 9:**
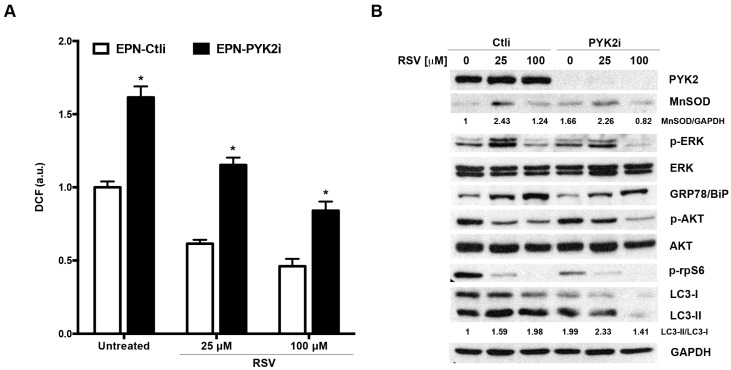
PYK2-depletion reproduces the effects of the PKM mutant in EPN cells: (**A**) Ctli and PYK2i EPN cells were treated for 24 h with RSV (25 and 100 µM), then ROS levels were estimated indirectly by measuring the fluorescence emitted by DCF. Data are mean ± SD of a representative experiment performed in triplicate. (*) indicates statistically significant differences (*p* < 0.05) between EPN-Ctli and EPN-PYK2i cells at each experimental point; (**B**) Total cell lysates from Ctli and PYK2i EPN cells in control conditions or treated with 25 and 100 µM RSV for 24 h were analyzed by Western blot using the indicated antibodies. GAPDH was used as loading control. The relative optical density of MnSOD levels normalized to GAPDH levels and of LC3-II/LC3-I ratio is shown.

To confirm with another approach the involvement of PYK2 in the regulation of ROS production, autophagy and sensitivity to RSV that we have observed comparing EPN and EPN-PKM cells, we knocked-down PYK2 expression in EPN cells by RNAi. EPN cells were transfected with a siRNAs pool targeting PYK2 and protein depletion was verified by Western blot (Figure 9B). As shown in Figure 9A, PYK2-depleted cells (PYK2i) display a ROS levels higher than control cells (Ctli), both in basal conditions and post-RSV treatment. Consistently with data obtained in EPN-PKM, we found that PYK2i displayed increased levels of MnSOD with respect to Ctli cells. In addition, RSV-induced increase of MnSOD expression is higher in Ctli than in PYK2i cells and that RSV increases MnSOD expression more at 25 µM concentration than at 100 µM (Figure 9B). Moreover, RSV treatment decreases AKT and rpS6 phosphorylation levels and increases GRP78/BiP expression and LC3-I > LC3-II conversion rate in a dose-dependent way in both in Ctli and PYK2i cells. However, LC3-II/LC3-I ratio is higher in PYK2i than in Ctli cells both in basal condition and after 25 µM RSV treatment (Figure 9B), suggesting that the depletion of PYK2 increases basal and RSV-induced autophagy as well as the expression of the PKM mutant. We detected very low levels of both LC3-I and LC3-II in PYK2i cells post 100 µM RSV treatment probably because the combination of PYK2-depletion and high dose RSV treatment increases the autophagy flux to the point that LC3-I is almost all converted in LC3-II that is, in turn, rapidly degraded into auto-phagolysosomes. Interestingly, the 25 µM RSV-induced increases of ERK phosphorylation levels observed in both EPN and EPN-PKM cells is almost abrogated in PYK2i cells, suggesting that this effect of RSV may involve PYK2 as a scaffold protein, even though it does not require its kinase activity. By contrast, 100 µM RSV decreases ERK phosphorylation in both Ctli and PYK2i, indicating that this effect is actually PYK2-independent. All together, these data confirm that the difference observed between EPN and EPN-PKM cells in response to RSV treatment are specifically due to PYK2 functional ablation.

## 3. Discussion

A detailed characterization of the effects of RSV on normal cells is required for a safe exploitation of the potentials of this molecule in humans, and long term (5–10 years) clinical trials are needed to prove the real chemo-preventive efficacy of RSV for human diseases [53]. In the present work, we report that RSV displays a strong bio-activity in EPN cells, a model of non-transformed prostate epithelial cells, modulating several cellular functions, also modulated by the non-receptor Tyr kinase PYK2 that emerges as a possible new mediator of RSV effects. Well aware of challenges and recommendation of “RSV 2012” and “RSV 2015” meetings, we have used concentrations of RSV both in the range achievable with diet (1 nM to 25 μM) and in the pharmacological one (50 to 100 μM) [48]. Analyzing the effects of RSV on EPN cells and their tumor-prone counterpart bearing a PYK2 dead kinase mutant (EPN-PKM), we found that RSV inhibits cell proliferation, decreases ROS levels and increases autophagy in both EPN and EPN-PKM cells. Interestingly, the expression of the PKM dead kinase mutant increases basal ROS production and autophagy levels and modulates the sensitivity of EPN cells to RSV effects. In EPN-PKM cells, the effects exerted by RSV on ROS and autophagy levels are different in intensity, compared to that observed in wild-type cells, suggesting that they combine with those of the PKM dead kinase mutant, which acts on the same cellular functions. In particular, our observations show that RSV canonically acts as an antioxidant agent in EPN and EPN-PKM cell, both via direct ROS scavenging, when added together with H_2_O_2_, and via increasing built-in antioxidant cellular defense. In that, we show that the expression of the PKM dead kinase mutant, as well as PYK2-knock down, increases basal levels of ROS production and MnSOD expression, and decreases the sensitivity to the antioxidant effect of RSV. In fact, post RSV treatment ROS levels remain higher in EPN-PKM and PYK2i than in the control counterparts. Furthermore, the MnSOD fold-change increase is lower in EPN-PKM and PYK2i with respect to the control counterparts. These data suggest that PYK2 is a player in the mechanisms regulating redox intracellular state in prostate cells and could influence the anti-oxidant action of RSV. In addition to this role in redox homeostasis, PYK2 ablation apparently correlates also with perturbation of autophagy. Autophagy, a self-degradative process providing energy sources, removing damaged organelles and misfolded proteins, plays a dual role: it is a surviving mechanism in stress conditions, such as nutrient starvation, oxidative and genotoxic stress or viral infections [54], but it can also lead to cell death when hyper-activated (called type II programmed cell death or autophagy cell death), thus possibly acting as an alternative to apoptosis [49]. EPN-PKM display a higher level of basal autophagy than EPN cells, as indicated by increased LC3-I > LC3-II conversion and enlargement of the autophagosomal/lysosomal compartment that are hallmarks of autophagy [55,56].

Consistently, PYK2i cells display increased LC3-II/LC3-I ratio and reduced rpS6 phosphorylation than Ctli cells, confirming the negative role of PYK2 in autophagy regulation. Despite several suggestive observations, the involvement of PYK2 in autophagy has not been clarified yet. FIP200, a known inhibitor of PYK2, is a regulatory partner of the ULKs–Atg13–FIP200 complex, which is essential for the induction of autophagy in mammalian cells [57]. Bae and coworkers reported that deletion of FIP200 resulted in multiple autophagy defects and increases cell death upon treatments with anticancer agents [58]. By contrast, the RSV ability to induce autophagy has been demonstrated in several cellular contexts, and seems due to its ability to directly inhibit mTOR through ATP competition [49]. Consistently, we have found that RSV increases autophagy in EPN and EPN-PKM cells. Interestingly, the effect of RSV is additive to that of the PKM mutant resulting in a more consistent LC3-I > LC3-II conversion in EPN-PKM than in EPN cells. As RSV is known to induce apoptosis mainly in cancer cells, we similarly propose that, because of the additive effect of RSV and PYK2 mutation or down-regulation, PYK2-defective prostate cancer cells may be more sensitive to RSV-induced type II cell death with respect to normal prostate cells, but further study are needed to confirm this hypothesis. Taken together, these results suggest that PYK2 could be an important mediator of intracellular redox state and of autophagy and reveal a functional interaction between RSV and PYK2 in EPN prostate cells, sustaining the idea that PYK2 may be a mediator of the complex mechanisms of action of RSV.

Besides the relationship between PYK2 and RSV, analyzing the effects of RSV on cell proliferation and survival in EPN, EPN-PKM and PC3 cells, we have pointed out the different sensitivity to RSV of non-transformed and cancer prostate cells. In fact, even though RSV induces growth arrest at all tested concentration in all tested cell lines, cell number remains approximately constant throughout the seven days of treatment in EPN cells, whereas it decreases in EPN-PKM and even more in PC3 cells. Since decrease in cell number is associated to apoptosis in PC3 but not in EPN-PKM cells, we can speculate that the reduction in cell number at the higher concentration of the phytoalexin in EPN-PKM cells may be explained by the consistent RSV-induced autophagy, suggesting a possible alternative “chemotherapeutic” action of RSV [59]. Moreover, even though with consistently different shades, the effects of RSV are reversible in all the three cell lines, since EPN, EPN-PKM and PC3 cells re-start to proliferate after RSV W/O. This latter observation is of particular importance for two opposite reasons. In fact, reversibility is good news for non-transformed EPN cells, possibly explaining low RSV toxicity reported in animal models and humans. The bad news is that RSV inhibitory effect is reversible also in tumor-prone EPN-PKM cells, and in metastatic PC3 cells, at least after 25 μM treatment. These observations are of interest in light of recent data showing a higher anti-cancer activity of low concentration of RSV [60]. Interestingly, even though long-term RSV treatment (seven days) points out a huge recovery difference between EPN and PC3 cells, it suggests that RSV may suffer the same “Achille’s heel” of most chemotherapeutic agents: the possibility that single cancer cells can survive and re-start proliferation also after long high-dose treatments.

Finally, we found that RSV-induced growth arrest is associated to remarkable morphological changes that revert after RSV W/O. In particular, we observed that 25 μM RSV increases cellular volume and complexity, and confers a “fried egg” shape to all the treated cells. On the other hand, 100 μM RSV-treated EPN and EPN-PKM cells “shrink” in a more “astrocytes-like” morphology, with volume and complexity lower that those observed in the 25 μM treated cells, but significantly higher than those observed in the untreated cells, whereas no significant dose-dependency was found for the RSV-induced morphological changes in PC3 cells. Notably, the observed changes in cellular shape and size, which are similar to those reported in RSV-treated HeLa, colon cancer [61] and ovarian cells [62], are apparently in contrast with the mTOR inhibition induced by RSV. In fact, since it is generally agreed that mTOR pathway positively regulating cell size [63], it is surprisingly that RSV can increase cellular dimension while inhibiting mTOR. The molecular mechanisms underlying this interesting phenomenon, as well as the functional consequences of RSV-induced morphological changes, represent an open question that should to be addressed.

All together, these data confirm canonically that RSV is able to induce apoptosis selectively in cancer cells, and that prostate cancer cells are much more sensitive to RSV anti-proliferative effects than non-transformed cells, enforcing the hypothesis that it could represent a low toxicity-chemotherapeutic agent. However, they shade also a new light on its preventive and protective effects because it cannot be excluded that long-term RSV treatment at low concentration may allow survival of tumor or tumor-prone cells. The observation that resveratrol has a strong bioactivity in normal cells and that its effects are reversible in tumor-prone and metastatic cells deserves further attention.

Considering the burden of health-care cost as a major global concern, dietary chemoprevention could provide an inexpensive, readily applicable and easily accessible approach and RSV is a good candidate. However, much work is needed before this molecule could be safely and efficiently recommended for humans. In fact, RSV targets and mechanisms of action have not been all identified so far, and the few small clinical studies published to date focus on therapeutic effects of RSV, obtained after short exposure to high concentrations. Clinical trials that address the disease-preventive potential of RSV, testing the effects of long-term treatments with low doses in both cellular and animal models, including those closer to humans than mice (i.e., pigs), are needed.

Moreover, to imagine translational applications of the relationship between RSV and PYK2 in prostate cells, the correlation between PYK2 status (i.e., expression levels, activity and mutations) and sensitivity to RSV in primary and immortalized human prostate cancer cell lines with different androgen sensitivity and degree of malignancy, and in animal models should be further investigated. Finally, the PYK2-RSV relationship could have interesting implications especially in light of recent data suggesting a protective role of oxidative stress in the development of metastasis [64,65].

## 4. Experimental Section

### 4.1. Chemicals

Chemicals were purchased from the following manufacturers: Dulbecco Modified Eagle’s Medium/HAM F12 from Gibco Cell Culture, Invitrogen Corporation, (Carlsbad, CA, USA); Penicillin, streptomycin, fetal calf serum (FCS), bovine serum albumin (BSA), and phosphate-buffered saline (PBS) from Eurobio (Les Ullis Cedex, France); RSV (#R5010) Sigma Aldrich (München, Germany); ECL System from Amersham Pharmacia (Buckinghamshire, UK), Bio-Rad assay and pre-stained protein standards from Bio-Rad (München, Germany); and Protease inhibitor cocktail tablets from Roche Diagnostics (Meylan, France).

### 4.2. Antibodies

Antibodies were purchased from the following sources: anti-MnSOD (#06-984) from Millipore (Merck Millipore, Billerica, MA, USA); anti-phospho-p44/42 MAP kinase (Thr202/Tyr204) (#9101), anti-p44/42 MAP kinase (#9102), anti-phospho-AKT (Thr308) (#9275), anti-AKT (#9272), anti-Caspase-7 (#9492), anti-Caspase-3 (#9665), anti-LC3A/B (#4108), anti-BIP(GRP-78) (#3177) and anti-PYK2 (3292S) from Cell Signaling Technology (distributed by Celbio S.p.A., Pero, Italy); anti-phospho S6 ribosomal protein (Ser240/244) (sc-397), PARP 1/2 H250 (sc-7150), anti-γ-Tubulin C11 (sc-17787) and anti-β-Actin I-19 (sc-1616) from Santa Cruz Biotechnology DBA (Milan, Italy); anti-α-Tubulin DM 1A (#T9026) from Sigma; anti-p27/Kip-1 (#610241) from BD Bioscience (Franklin Lakes, NJ, USA); anti-GAPDH (6C5) (ab8245) from Abcam (Cambridge, UK); and anti-rabbit IgG, anti-mouse IgG and anti-goat IgG from Amersham Pharmacia (Buckinghamshire, UK).

### 4.3. Cell Culture and Growth Curve

EPN and EPN-PKM cells, originally obtained in our lab as previously described [25,32], are routinely cultured in DMEM/F12 supplemented with 3% FBS (standard medium). PC3 cells are routinely cultured in RPMI medium supplemented with 5% FBS. For proliferation assay, EPN, EPN-PKM and PC3 cells are seeded in 60 mm culture dishes in standard medium or in the presence of different concentrations of RSV (25 to 100 µM) as detailed in the figure legend. Triplicate dishes per each experimental time point are trypsinized and cell number is determined by counting cell suspension in a Neubauer hemocytometer. The values reported represent the mean ± SD of three independent samples per each experimental point.

### 4.4. Cristal Violet Staining

Colony arising from wash-out experiments were stained with Crystal Violet. Briefly, cells were washed with PBS, the cells attached to the bottom of the plate were fixed and stained with 0.4% crystal violet solution in methanol for 30 min, then extensively washed with water, dried and photographed with Canon GC5 camera (Canon Italia S.p.A, 20063 Cernusco sul Naviglio, Milan, Italy).

### 4.5. Soft Agar Assay

Soft agar Assay was performed as follows 6 cm diameter cell culture dishes of were layered with 3 mL of medium with 0.4% soft agar. EPN, EPN-PKM and PC3 cells, as positive control, cultured in standard conditions were trypsinized, centrifuged and re-suspended in a single-cell suspension of 75,000 viable cells/mL. The latter was mixed with complete medium containing 0.4% soft agar at a ratio 1:2. The cell suspensions were seeded onto the Petri dishes containing the solidified agar medium, 1 mL/dish, and incubated at 37 °C and 5% CO_2_. Cultures were observed under microscope just after plating, to verify the absence of cell aggregates, and next periodically checked for colonies formation. After two weeks, the colonies were photographed.

### 4.6. Measurement of ROS

The formation of ROS was evaluated by means of the probe 2′,7′-dichlorofluorescin-diacetate (H2DCF-DA, Sigma-Aldrich). Briefly, 2 × 10^4^ EPN, EPN-PKM and PC3 cells/well have been seeded in 24-well plates for 24 h. Fluorometric determination of intracellular ROS was estimated by loading the cells with H2DCF-DA (10 µM) for 45 min at 37 °C. Cells have been washed twice with PBS buffer, RSV and H_2_O_2_ were added at appropriate concentration and plates were placed in a fluorescent microplate reader (Perkin Elmer LS 55 Luminescence Spectrometer, Perkin-Elmer Ltd., Beaconsfield, UK). To study the effect of RSV pre-treatment on H_2_O_2_-induced ROS production, cells were maintained in the presence of RSV for 24 h, then washed twice with PBS buffer, overloaded with H2DCF-DA (10 µM) for 45 min at 37 °C, washed twice with PBS buffer, then H_2_O_2_ was added and plates were placed in a fluorescent micro-plate reader. Fluorescence have been monitored using an excitation wavelength of 485 nm and an emission wavelength of 538 nm. Each experiment has been performed at least three times in triplicates.

### 4.7. Western Blotting

Cells were grown to sub-confluence in standard medium or serum-starved for 48 h and treated as indicated. Cells were harvested in lysis buffer (50 mM HEPES, 150 mM NaCl, 1 mM EDTA, 1 mM EGTA, 10% glycerol, 1% Triton-X-100, 1 mM phenylmethylsulfonyl fluoride, protease inhibitor cocktail tablet, 0.5 mM sodium orthovanadate, 20 mM sodium pyrophosphate). The lysates were incubated for 30 min on ice, and supernatants were collected and centrifuged for 10 min at 14,000× *g*. Protein concentration was estimated by a modified Bradford assay and 25 or 50 μg/lane of total proteins were separated on SDS gels and transferred to nitrocellulose membranes [66]. Membranes were treated with a blocking buffer (25 mM Tris, pH 7.4, 200 mM NaCl, 0.5% Triton X-100) containing 5% non-fat powdered milk for 1 h at room temperature. Incubation with the primary antibody was carried out overnight at 4 °C. After serial washings, membranes were incubated with the horseradish peroxidase-conjugated secondary antibody for 1 h at room temperature. Following further washings of the membranes, chemiluminescence was generated ECL system. Densitometric analysis were performed by using the NIH Image software (Bethesda, MD, USA).

### 4.8. Flow Cytometry

Cells were washed two times after stimulation with RSV, resuspended in PBS, read at cytoflurimeter (BD Facs Canto II, BD Biosciences, San Jose, CA, USA) and evaluated for their FSC and SSC parameters.

### 4.9. Fluorescence Microscopy

Lysotracker and monodansylcadaverin were used to label lysosomes and autophagosomes, respectively. Briefly, cells grown on coverslips were incubated with Lysotracker probe (Molecular Probes, Arese, Milan, Italy) for 1 h at 37 °C then washed with PBS, fixed with 4% PFA, quenched with 50 mM NH4Cl. Cells grown on bottom-glass dishes were incubated with 50 mM monodansilcadaverin (Sigma-Aldrich) in PBS for 10 min at 37 °C and imaged in vivo in PBS.

Microtubules were stained by using an antibody against alpha-tubulin (Sigma-Aldrich) detected with FITC-conjugated secondary antibodies (Jackson ImmunoResearch Laboratories, Inc., West Grove, PA, USA), actin with TRITC-conjugated phalloidin (Sigma-Aldrich, Saint Louis, MO, USA).

Images were collected using a laser scanning microscope (LSM 510 META, Carl Zeiss Microimaging, Inc., Jena, Germany) equipped with a planapo 63x oil-immersion (NA 1.4) objective lens. All image processing was done using LSM 510 software.

### 4.10. RNA Intereference

RNA interference was obtained using a siRNAs pool specific for human PTK2B (ON-TARGETplus SMARTpool, Human PTK2B (L-003165-00) (Thermo Scientific Dharmacon, Arese, Milan, Italy). ON-TARGETplus Control pool, Non-targeting pool (D-001810-10-05) (Thermo Scientific Dharmacon) was used as negative control. Cells were transfected with 25 nM of specific or control siRNAs pool using RNAiMAX reagent (Invitrogen) as previously described [67]. Treatment with RSV started 48 h post-transfection.

### 4.11. Statistical Analysis

Statistical analysis was performed using Student’s *t*-test to compare two sets of data and Anova with Bonferroni’s correction to test continuous variables. *p*-Value < 0.05 were considered statistically significant.

## Appendix A

**Table A1:** Statistical analysis was performed by Anova followed by Bonferroni post-test, as shown in table. (***) indicates statistically significant differences (*p* < 0.001).

One-Way Analysis of Variance						
	EPN		EPN-PKM		PC3	
Hours of NAC treatment	48	72	48	72	48	72
*p*-Value	<0.0001	<0.0001	<0.0001	<0.0001	<0.0001	<0.0001
*p*-Value summary	***	***	***	***	***	***
α *p* < 0.05	Yes	Yes	Yes	Yes	Yes	Yes
*F*	26.06	41.51	10.82	22.09	19.3	17.41
*R* square	0.7363	0.8164	0.537	0.703	0.6819	0.6511
